# Protein QID74 protects the cell wall of *Trichoderma* from degradation caused by its own chitinase, which lacks a carbohydrate-binding module

**DOI:** 10.1128/mbio.00018-26

**Published:** 2026-04-20

**Authors:** Jun-Jin Deng, Zhao-Fu Chen, Ming-Shu Zhang, Jing Liu, Zhi-Lin Wang, Jia-Zhou Li, Shaowen Wu, Xiao-Chun Luo

**Affiliations:** 1 Agro-Biological Gene Research Center, Guangdong Key Laboratory for Crop Germplasm Resources Preservation and Utilization, Guangdong Academy of Agricultural Sciences 608228 , Guangzhou, Guangdong, China; 2 Guangdong Laboratory for Lingnan Modern Agriculture Heyuan Sub-Center, Heyuan, Guangdong, China; 3 School of Biology and Biological Engineering, South China University of Technology 199191 , Guangzhou, Guangdong, China; Universidade de Sao Paulo Campus de Ribeirao Preto, Ribeirao Preto, Sao Paulo, Brazil

**Keywords:** fungal cell wall, *Trichoderma*, chitinases, self-protection, carbohydrate-binding module

## Abstract

The fungal cell wall, formed by a chitin backbone, is critical for cellular integrity and environmental fitness. Mycoparasitic fungi of the genus *Trichoderma* deploy an arsenal of potent chitinases to dismantle the cell walls of prey fungi. However, the mechanisms that safeguard the mycoparasite from self-inflicted enzymatic damage remain poorly understood. While most chitinases possess carbohydrate-binding modules (CBMs) to facilitate substrate recognition, the critical group A chitinase of *Trichoderma harzianum* is conspicuously CBM-free. In this study, we investigated the evolutionary and functional rationale for this structural omission by comparing native Chit46 with engineered CBM-fused variants. Results demonstrate that while native Chit46 promotes the hyphal growth of *T. harzianum*, the fusion of a CBM shifts the enzymatic activity from highly specific to self-destructive. CBM-fused variants significantly enhanced the degradation of host cell walls but simultaneously triggered the hydrolysis of *Trichoderma*’s own hyphae, a phenotype absent in the native enzyme. These findings suggest an evolutionary selection against CBMs in specific chitinases to prevent deleterious autocatalytic activity. Furthermore, QID74 was identified as a critical cell wall-associated “shield” protein that specifically binds Chit46 to prevent self-hydrolysis. Structural characterization revealed that QID74 is composed of 13 tandem 59-residue repeats containing conserved tridecapeptide motifs (NGKQCVCPKGQVW). Biochemical validation via His pull-down and isothermal titration calorimetry confirmed that these repetitive units are essential and sufficient for chitinase binding. Our results illuminate a sophisticated “arrow-and-shield” mechanism that balances offensive mycoparasitic capabilities with the maintenance of self-integrity, providing new insights into fungal self-recognition and the optimization of microbial biocontrol agents.

**IMPORTANCE:**

This study uncovers a unique survival strategy in *Trichoderma*. By lacking a carbohydrate-binding module, *Trichoderma*’s chitinase can effectively degrade host cell walls while minimizing self-damage. The protective protein QID74 further safeguards the cell wall by binding to the chitinase. This research offers novel insights into fungal biology and ecology, with potential applications in developing sustainable biocontrol agents.

## INTRODUCTION

The fungal cell wall is a dynamic and critical structural component serving essential functions, including protection, maintenance of cell viability, morphogenesis, and pathogenicity. Its composition and architecture are precisely regulated in response to environmental stimuli, profoundly influencing ecological interactions. Understanding the mechanisms governing the synthesis and degradation of this primary barrier is crucial for elucidating fungal behavior, yet these processes remain incompletely understood.

Among diverse fungal species, mycoparasites, such as species of the genus *Trichoderma*, provide powerful models for studying cell wall recognition and degradation. These fungi possess remarkable capability to lyse the cell walls of host fungi while simultaneously avoiding self-damage, thereby offering vital insights into the intricate mechanisms underpinning mycoparasitism (1). During invasive processes, *Trichoderma* mycelium actively grows toward the host, forming extensive coils and secreting an arsenal of hydrolytic enzymes evolved to effectively degrade the host cell wall (2). Among these, chitinases are the major determinants for successful mycoparasitism, and differences between mycoparasitic and saprophytic species often stem from a diversification of enzymes involved in chitin metabolism (3). The architectural scaffold of fungal cell walls predominantly comprises chitin and β-1,3-glucan. Chitin microfibrils, located in the inner layers of the cell wall, are fundamental to maintaining structural integrity and mechanical resilience (4), forming a crucial barrier against hostile environmental factors and enzymatic attack.

Chitin constitutes 10%–15% of the cell wall in filamentous ascomycetes, correlating with a diverse array of chitin synthases and chitinases detected in their genomes (5), especially within the genus *Trichoderma* (6). Beyond their roles in mycoparasitism and nutrient acquisition, chitinases are crucial for autolysis, age-related cell wall recycling, and growth-dependent remodeling processes (7). Despite their vital importance, the multifaceted mechanisms governing their actions remain poorly defined. Critical questions arise: how do healthy *Trichoderma* actively degrade host chitin while preventing self-damage from their own potent chitinases during mycoparasitic attacks? What mechanisms enable these chitinases to distinguish self- from non-self-cell walls and accurately target the appropriate sites of action? Addressing these questions is essential for advancing our understanding of fungal biology and mycoparasitism.

Fungal chitinases predominantly belong to glycoside hydrolase (GH) family 18. *Trichoderma* GH18 chitinases are classified into three groups: A, B, and C (8). Group A chitinases are particularly noteworthy as they lack carbohydrate-binding modules (CBMs), whereas groups B and C possess CBMs at their C- or N-termini, respectively. CBMs are independent non-catalytic domains essential for substrate recognition and specific binding in many chitinases (9). Indeed, artificial CBM-fused chitinases originating from mycoparasites often showed more potent antifungal activities (10–12), and recombinant *T. harzianum* expressing CBM-fused CHIT42 exhibited stronger inhibition of phytopathogenic fungi (10, 13, 14). Specific group A chitinases, such as ECH42, have been implicated in exogenous chitin degradation, host cell wall attack, and self-cell wall autolysis (15–17). It is intriguing that mycoparasitic fungi like *Trichoderma* primarily depend on CBM-free chitinases for these major roles, unlike other microorganisms where CBMs are prevalent and vital for chitinase efficacy. This phenomenon suggests a potential evolutionary adaptation facilitating effective degradation of external chitin sources while minimizing self-recognition that could trigger deleterious autolysis. In this context, it is hypothesized that *Trichoderma* regulates substrate accessibility to protect its own cell wall (18), potentially through the upregulation of chitin deacetylase, which aids in converting chitin to chitosan (19). In *Trichoderma*, carbohydrate-binding proteins, along with hydrophobins, have emerged as crucial components of this protective mechanism (6, 20). Cell wall protective proteins, such as the secreted cysteine-rich repeat protein (SCREP) QID74, have emerged as crucial components of this protective mechanism (21). Deletion of QID74 in *Trichoderma* results in increased sensitivity to exogenous lytic enzymes (22). Besides, QID74 was suggested to exhibit functionalities similar to carbohydrate-binding proteins by interacting with short oligosaccharides. This interaction could, in turn, influence the induction of cell wall-degrading enzymes or potentially bind directly to chitin, thereby masking it from enzymatic degradation (18). However, direct biochemical evidence explaining how QID74 protects the cell wall and whether it interacts with chitin or chitinases is still lacking.

Chit46 (also known as CHIT42, ECH42, CHI18-5, or ECH1) belongs to group A and is a major contributor to chitinase activity in *Trichoderma* (23). We previously found that chitin encapsulated by proteins in shrimp shells is resistant to hydrolysis by chitinases (24), suggesting a protective mechanism analogous to that proposed for fungal cell walls. Therefore, three chimeric chitinases were constructed by fusing CBM to Chit46 (25). These variants, which displayed enhanced enzymatic activities against pure substrates, provide a reverse model to understand why many fungal chitinases naturally lack CBMs. In this study, we aim to elucidate the interactions of chitinases with self- versus host cell walls. The effects of exogenous Chit46 and its CBM-fused variants on the growth and morphology of *T. harzianum* and its host were analyzed. Furthermore, recombinant truncated QID74 was constructed to determine if it can directly inhibit the chitinolytic activity of Chit46. The interaction between QID74 and Chit46 was investigated by His pull-down and isothermal titration calorimetry (ITC) assay. This study discusses the mechanisms through which CBM-lacking chitinases enable *Trichoderma* to differentiate between self- and non-self-substrates and how they interact with protective proteins to maintain overall cell wall architecture and resilience during mycoparasitic attacks.

## MATERIALS AND METHODS

### Materials, enzymes, and reagents

The fungal strains *T. harzianum* GIM 3.442, *Botrytis cinerea* GIM 3.47, *Mucor circinelloides* GIM 3.79, *Rhizoctonia solani* GIM 3.512, and *Aspergillus fumigatus* GIM 3.20 were obtained from the Guangdong Microbial Culture Collection Center. Recombinant chitinase Chit46 and its engineered variants—Chit46-CBM3, Chit46-CBM6, and Chit46-CBM26—were produced and purified as previously described (25, 26). The specific activities of the enzymes were determined to be 34.5 U/mg for Chit46, 75.8 U/mg for Chit46-CBM3, 47.0 U/mg for Chit46-CBM6, and 38.8 U/mg for Chit46-CBM26. Kinetic analysis using colloidal chitin as a substrate yielded Michaelis constants (*K_m_*) of 0.65 g/L for Chit46, 0.21 g/L for Chit46-CBM3, 0.35 g/L for Chit46-CBM6, and 0.30 g/L for Chit46-CBM26.

### Antifungal activity against phytopathogenic fungi

The effect of Chit46 and the chimeric chitinases on the growth of phytopathogenic fungi was investigated following the previous study (27). Purified chitinases were filtered (0.22 μm) and added into PDA medium just prior to pouring plate. Heat-denatured chitinase served as a negative control. A 1 μL spore suspension (10^6^/mL) of the target phytopathogen was inoculated at the center of each plate, which was then incubated at 25°C. Colony diameters were measured daily. After several days of cultivation, the percentage of mycelial growth inhibition (GI) was evaluated using the following formula:


GI=(R−r)/R×100%,

where *R* and *r* refer to the average diameters of fungal mycelia on control and experimental plates, respectively. Each treatment was conducted in triplicate and repeated thrice. The ED_50_ of chitinases on *B. cinerea* was analyzed and conducted using GraphPad Prism 7.

### Effect of chitinases on *T. harzianum* growth and morphology

To determine the effect of exogenous chitinases on *T. harzianum*, the fungal colony growth assay described above was performed using PDA medium supplemented with 0.1 U/mL of the respective chitinase. After incubation, mycelia and spores were harvested from the plates using 2 mL of sterile water. Mycelial morphology was observed using an optical microscope. Spore concentration was determined using a hemocytometer.

### Effect of chitinases on the hyphae and spores of *T. harzianum*

For analysis of hyphal integrity, 100 mg of *T. harzianum* mycelia was collected from a 3-day-old PDA plate and treated with 20 U of the specified chitinase. Heat-denatured Chit46 served as a control. After incubation at 25°C for 12 h, samples were fixed in 2.5% glutaraldehyde and observed via optical and scanning electron microscope (SEM). For analysis of conidia integrity and germination, spores were harvested by flooding a 7-day-old *T. harzianum* PDA plate with sterile water. The resulting conidia suspension was adjusted to a concentration of 10⁶ conidia/mL. The suspension was incubated with 10 U of chitinase in an equal volume of PDB at 25°C for 12 h. Conidia germination was assessed microscopically. Each treatment was performed in triplicate, and the entire experiment was repeated twice.

To analyze enzyme activity on isolated cell walls, fungal cell walls were prepared from *T. harzianum* mycelia according to an established protocol (28). Prior to enzymatic treatment, the cell wall material was sonicated using a XM-650DT Sonifier (Jiangsu, China) to ensure uniform suspension and increased substrate accessibility.

### Recombinant expression and purification of truncated QID74

The DNA fragments encoding the truncated QID74 (GenBank accession no. X95671.1) were amplified by PCR using *T. harzianum* cDNA as the template and the specific primers listed in Table S1. The resulting PCR products were purified and cloned into pET-28a(+) using ClonExpress Ultra One Step Cloning Kit (Vazyme, Nanjing). Recombinant plasmids were transformed into *Escherichia coli* BL21(DE3). Positive transformants were cultivated at 37°C for 4–6 h, and then protein expression was induced by 0.2 mM IPTG at 37°C for 3 h. The recombinant truncated QID74 protein was purified by nickel-affinity chromatography.

### Bioinformatic and structural analysis

The three-dimensional structures of QID74, Chit46, and the fusion proteins were predicted using AlphaFold2. The predicted models were subjected to energy minimization using the CHARMM36 force field in GROMACS to optimize local structural geometry. Protein–protein interactions were analyzed using the AlphaFold Server (29) and PDBePISA (https://www.ebi.ac.uk/pdbe/pisa/pistart.html). Repetitive sequences within QID74 were identified and analyzed by BioAider (30), and sequence logos were generated using WebLogo (31). Maximum likelihood phylogenetic trees were constructed using Mega7.0 (32) and visualized using iTOL (33).

### Chitin-binding and enzyme inhibition assays

The chitin-binding capacity of truncated QID74 was determined for 0.2 g chitin powder or colloidal chitin by mixing 20 mL of purified protein (40 mg) with either 0.2 g of chitin powder or colloidal chitin. The mixture was incubated at 25°C for 10 min with gentle agitation. Following centrifugation, the protein concentration in the supernatant was determined using a bicinchoninic acid Protein Assay Kit (Sangon, Shanghai, China). The amount of adsorbed protein was calculated by subtracting the protein remaining in the supernatant from the initial amount of protein added.

To evaluate the effect of QID74 on chitinase activity, Chit46 or CBM-fused chitinases were pre-incubated with an equimolar amount of truncated QID74 protein at room temperature for 10 min. Chitinase activity was then assayed using chitin powder as the substrate. Kinetic parameters (*K_m_* and *V*_max_) were determined by measuring chitinase activity toward varying concentrations of colloidal chitin (0.01–10 g/L). Data were fitted to the Michaelis–Menten equation using non-linear regression analysis in Origin 9.1.

### His pull-down assay

The Chit46 without a His-tag was prepared by a cleavable self-aggregating tag 2.0 method (34). Purified His-tagged repeat-de protein was incubated with pre-equilibrated cobalt resin in binding buffer (50 mM Tris-HCl, pH 8.0, 300 mM NaCl, and 10 mM imidazole) for 1 h at 4°C. The resin was then washed extensively with wash buffer (50 mM Tris-HCl, pH 8.0, 300 mM NaCl, and 20 mM imidazole) to remove non-specifically bound proteins. Purified Chit46 protein (without a His-tag) was then added as the prey and incubated with the bait-bound resin for 12 h at 4°C. Following incubation, the resin was washed again to remove unbound Chit46. Specifically bound protein complexes were eluted using elution buffer containing 250 mM imidazole. Eluates were resolved by SDS-PAGE and visualized by Coomassie blue staining. Controls included resin incubated with Chit46 only (no bait) and resin bound with repeat-de protein only (no prey).

### ITC binding assay

The molecular interaction between Chit46 and the repeat-de protein was characterized using a Nano ITC isothermal titration calorimeter (TA Instruments). Prior to the experiment, all protein solutions were degassed under vacuum for 10 min. The sample cell was filled with 300 µL of Chit46 at a concentration of 2.5 µM. The titration syringe was loaded with 60 µL of the repeat-de protein at a concentration of 25 µM. Titration was performed at 25°C by making 20 successive injections of the repeat-de protein into the Chit46 solution. Each injection was followed by a 200-s equilibration interval. Thermodynamic parameters, including the equilibrium dissociation constant (*K*_*D*_), stoichiometry (*N*), enthalpy (Δ*H*), and entropy (Δ*S*), were calculated from the integrated heat data using NanoAnalyze software.

## RESULTS AND DISCUSSION

### CBM-fused chitinases exhibit stronger inhibition against fungal phytopathogens

**Fig 1:**
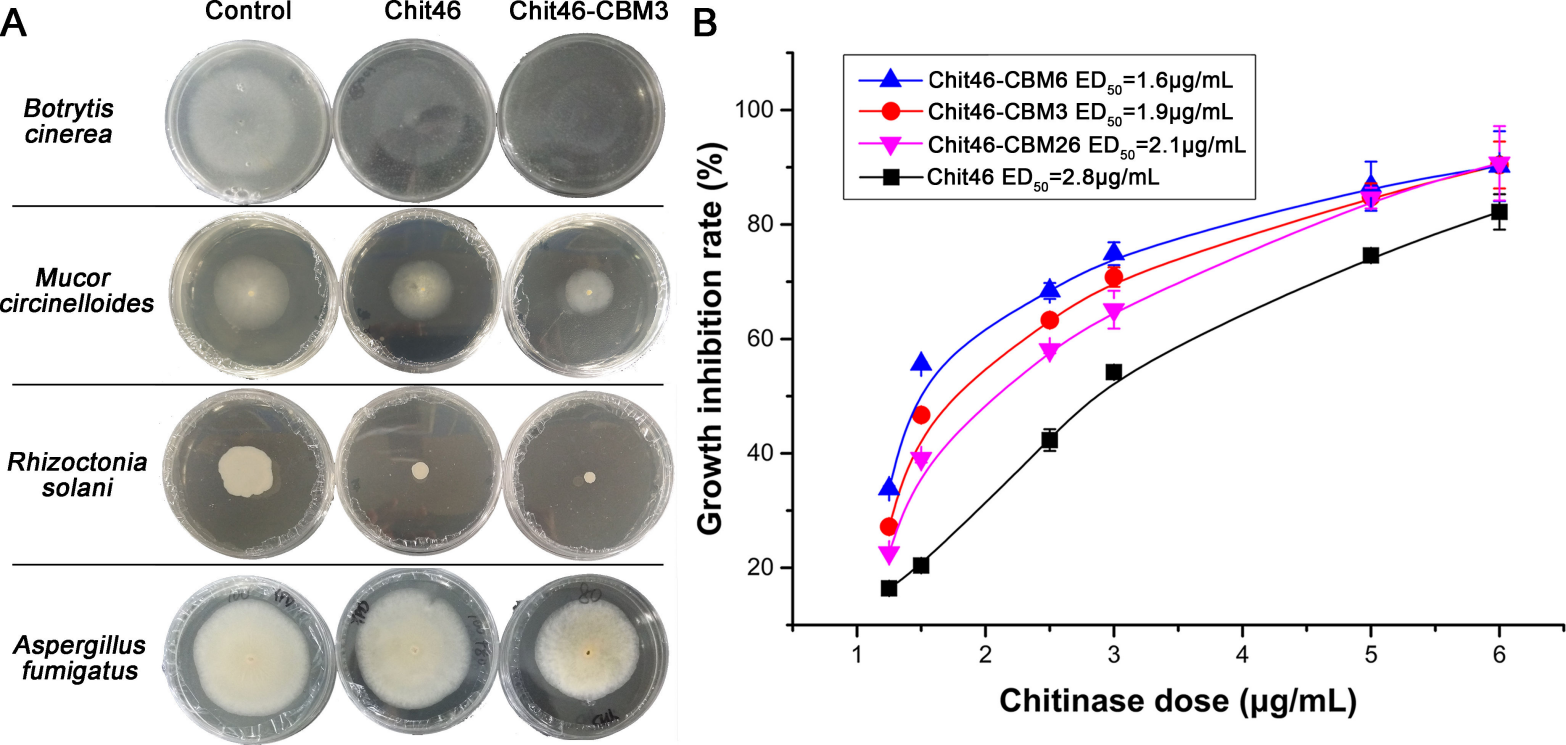
The antifungal activities of Chit46 and chimeric chitinases. (**A**) Antifungal activities of chitinases on *Botrytis cinerea*, *Mucor circinelloides*, *Rhizoctonia solani*, and *Aspergillus fumigatus*. (**B**) ED_50_ analysis of four chitinases on *B. cinerea*. Each plate was centrally inoculated with 1 μL of a fresh *B. cinerea* conidial suspension (~10^6^ conidia/mL). The plates were incubated at 25°C for 48 h, a time point chosen to allow sufficient fungal growth in control plates without reaching the plate edge. Radial growth was measured using digital calipers in two perpendicular directions to calculate the average diameter. The plates were incubated at 25°C for 48 h, a time point chosen to allow sufficient fungal growth in control plates without reaching the plate edge. Radial growth was measured using digital calipers in two perpendicular directions to calculate the average diameter.

Chitinases are potent effectors in mycoparasitic interactions, playing a key role in host cell wall degradation. To investigate the impact of CBM fusion on the antifungal activity of Chit46 against fungal phytopathogens, we performed growth inhibition assays on PDA plates containing the purified enzymes. We observed significant growth inhibition of *B. cinerea*, *M. circinelloides*, *R. solani*, and *A. fumigatus* in the presence of both native and CBM-fused Chit46 (Fig. 1A). As hypothesized, fusion with CBMs significantly enhanced the antifungal potency of Chit46. Specifically, Chit46-CBM3 exhibited stronger inhibition against all four pathogens tested, with GI values of 34.4% ± 0.4%, 38.5% ± 0.9%, 78.5% ± 6.2%, and 13.7% ± 0.3%, respectively. In comparison, native Chit46 showed GI values of 15.0% ± 0.1%, 17.8% ± 0.3%, 66.2% ± 4.2%, and 0.0% ± 0.1%, respectively. Furthermore, dose–response analysis revealed that Chit46 fused with CBM6, CBM3, and CBM26 displayed lower ED_50_ against *B. cinerea* (1.6, 1.9, and 2.1 μg/mL, respectively) compared to native Chit46 (2.8 μg/mL) (Fig. 1B), confirming their superior antifungal efficacy.

Fungal infections pose a significant global challenge to both plant health and human well-being. *T. harzianum* is well regarded for its antagonistic properties and is widely used as a biological control agent, offering an alternative to chemical pesticides for managing fungal diseases. The biocontrol efficacy of *Trichoderma* is attributed, in part, to the action of chitinases and proteases that degrade host cell wall components, such as chitin and proteins (2, 35). Given that chitin is absent in vertebrates and higher plants, chitinases present a promising target for the development of biopesticides and therapeutic agents. Among these, Chit46 has been extensively utilized in transgenic plants—such as apples, tobacco, and cotton—to confer resistance against fungal diseases (36). In this study, we demonstrated that CBM-fused Chit46 variants possess significantly enhanced antifungal activities compared to the native enzyme. These findings suggest that CBM-fused chitinases have the potential to serve as superior biocontrol agents for managing pathogenic fungi in agriculture and food safety applications.

### Exogenous Chit46 promotes, while CBM-fused variants inhibit, the growth and development of *T. harzianum*

**Fig 2:**
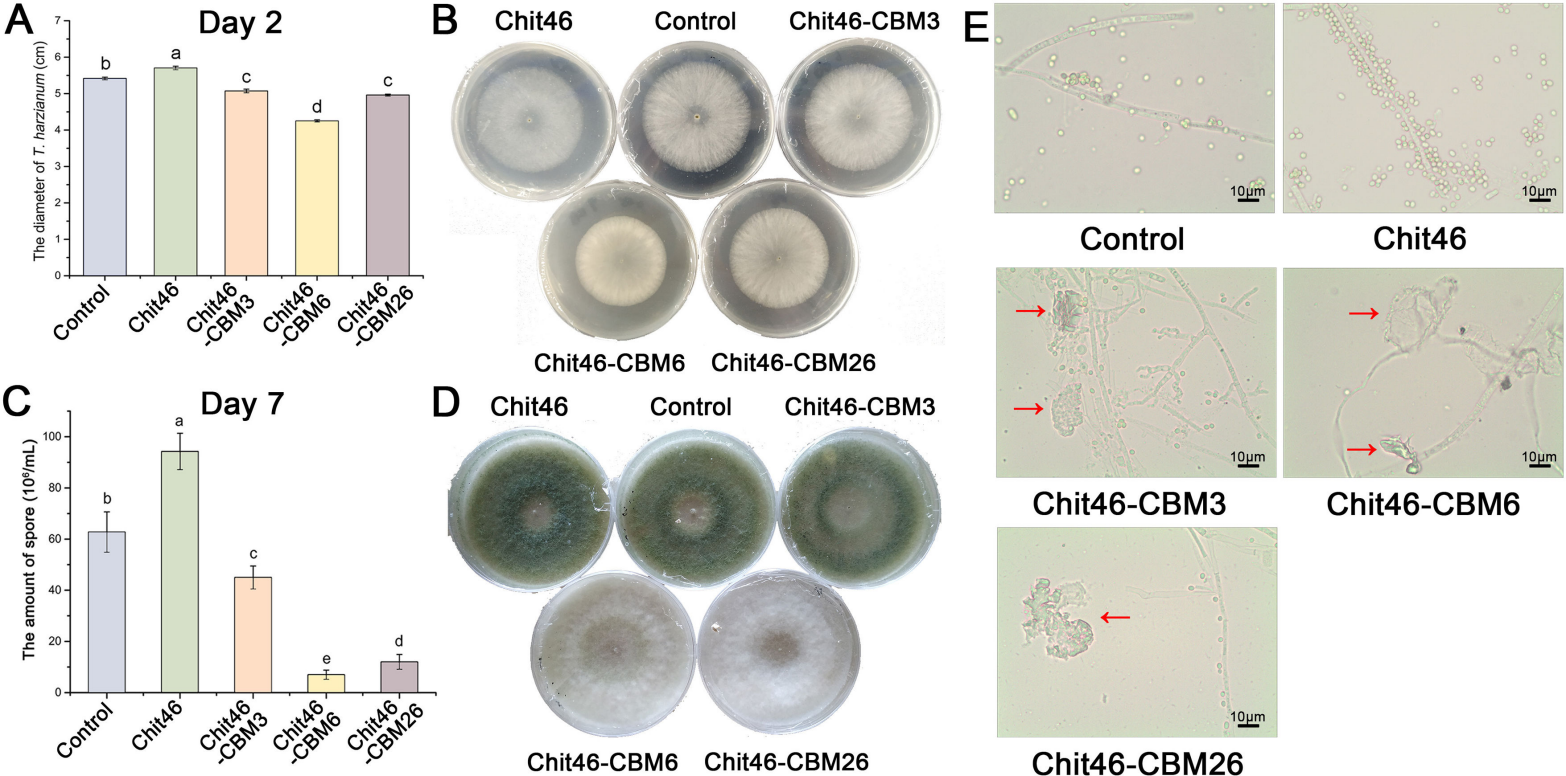
The effects of chitinases on the growth of *Trichoderma harzianum*. Each treatment was conducted in triplicate and repeated thrice. (**A**) The diameter of *T. harzianum* on chitinase-containing plates at day 2. (**B**) *T. harzianum* mycelia on chitinase-containing plates at day 2. (**C**) Number of conidia at day 7. (**D**) *T. harzianum* mycelia on chitinase-containing plates at day 7. (**E**) Light micrograph of *T. harzianum* mycelia at day 7. Red arrows indicate the damaged hypha.

To investigate the functional implications of the natural lack of CBMs in group A *Trichoderma* chitinases, we analyzed the effects of exogenously applied native Chit46 and three engineered CBM-fused variants on the growth and development of wild-type *T. harzianum*. We first examined the impact on mycelial growth. As shown in Fig. 2A and B, the addition of native Chit46 to the culture medium significantly promoted the radial growth of *T. harzianum*, resulting in larger colony diameters compared to the control at day 2. In stark contrast, the addition of chimeric chitinases inhibited growth, with Chit46-CBM3, Chit46-CBM6, and Chit46-CBM26 causing growth inhibition ratios of 6.3%, 21.5%, and 8.4%, respectively. Further observations after a 7-day incubation revealed striking differences in colony morphology and pigmentation. Control cultures and those treated with native Chit46 developed a characteristic dark green coloration, indicative of profuse conidiation (37). Quantification of spore production confirmed these observations: cultures treated with Chit46-CBM6 and Chit46-CBM26 yielded significantly fewer spores, while Chit46-CBM3 caused an intermediate reduction (Fig. 2C). The green pigmentation appeared even more intense in Chit46-treated cultures, suggesting a potential enhancement of conidia production (Fig. 2D). Conversely, cultures treated with CBM-fused variants showed significantly reduced pigmentation. The green color was noticeably lighter in the Chit46-CBM3 treatment group, while cultures treated with Chit46-CBM6 or Chit46-CBM26 remained predominantly white, indicating a severe suppression of conidia formation.

Microscopic examination confirmed these macroscopic observations (Fig. 2E). Cultures treated with native Chit46 displayed abundant conidia. In contrast, cultures exposed to CBM-fused chitinases, particularly Chit46-CBM6 and Chit46-CBM26, showed a scarcity of conidia. Furthermore, preliminary microscopic analysis revealed morphological anomalies and signs of lysis in hyphae treated with the CBM-fused enzymes, suggesting potential structural compromise. These observations indicate that while exogenous native Chit46 supports the growth and development of *T. harzianum*, CBM-fused variants exert deleterious effects, inhibiting growth and causing hyphal injury. However, the observed hyphal damage is based on limited microscopic sampling from plate cultures, where contact between the exogenously applied enzymes and the fungal mycelium may be spatially restricted. Therefore, these indications of structural damage should be interpreted as preliminary; more detailed analyses are necessary to confirm the extent and mechanism of hyphal impairment.

Mycelial growth rate and spore production are widely recognized as key fitness metrics for filamentous fungi (38). Our results demonstrate that exogenous native Chit46 enhances these fitness parameters in *Trichoderma*, whereas CBM-fused variants reduce them. Interestingly, while mechanical injury to hyphae typically induces conidiation in *Trichoderma* as a stress response (39, 40), the hyphal damage caused by CBM-fused chitinases in our study was associated with the inhibition of conidiation. This suggests that chitinase-induced damage may trigger distinct physiological responses or signaling pathways compared to mechanical stress, leading to the suppression rather than induction of conidial development.

### Exogenous CBM-fused chitinases damage *T. harzianum* hyphae and germinating conidia

**Fig 3:**
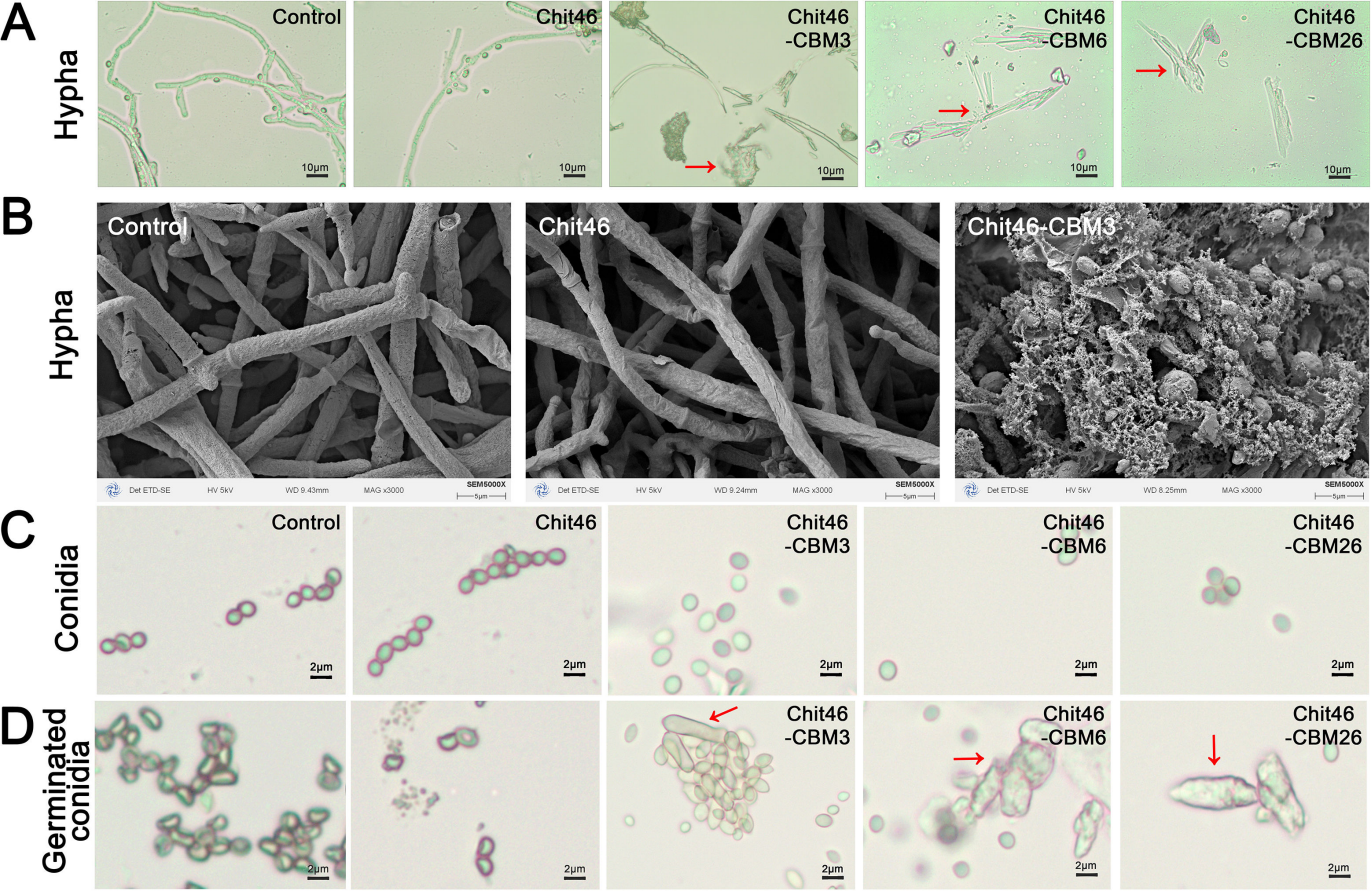
The effects of chitinases on *Trichoderma harzianum*. Red arrows indicate the damaged hypha. (**A**) Optical microscopy observation of hypha. (**B**) Scanning electron microscope observation of hypha. (**C**) Optical microscopy observation of conidia. (**D**) Optical microscopy observation of geminated conidia.

To investigate if the observed inhibition of *T. harzianum* growth and conidiation was a direct consequence of enzymatic attack by CBM-fused chitinases, we examined the morphology of mycelia and conidia following treatment with native Chit46 and CBM-fused variants. Control hyphae displayed normal, smooth surface structures under both optical and SEM, indicative of healthy growth (Fig. 3A and B). Similarly, treatment with native Chit46 did not induce any noticeable alterations in hyphal morphology. In stark contrast, exposure to CBM-fused chitinases resulted in pronounced morphological disruptions. While Chit46-treated hyphae maintained a consistent diameter and general morphology comparable to the control, SEM analysis revealed that hyphae exposed to Chit46-CBM3 exhibited severe structural irregularities, including the presence of perforations, crevices, and complete hyphal breakage (Fig. S1). This damage was corroborated by enzymatic assays using isolated *T. harzianum* cell walls, which revealed that CBM fusion markedly enhanced chitinase hydrolytic activity, with Chit46-CBM3 exhibiting a fourfold increase over native Chit46 (Fig. S2). The extensive degradation of the vegetative mycelium likely underlies the observed dual inhibition of both radial expansion and conidiation. The fungal cell wall integrity pathway plays a critical role in the early phases of asexual development (41); its disruption via enzymatic cell wall damage would be expected to impair conidiogenesis and spore maturation.

We also examined the effects of these chitinases on *T. harzianum* conidia. Mature, dispersed conidia appeared resistant to enzymatic attack, maintaining their structural integrity with no significant morphological differences observed among treatment groups (Fig. 3C). This suggests that the cell walls of mature conidia are more resistant to degradation by CBM-fused chitinases compared to hyphal cell walls, likely due to differences in composition, such as a higher proportion of melanin relative to chitin in conidial walls (42, 43). Heavily pigmented conidia are known to play a critical role in fungal survival and protection against various stressors (44). However, germinating conidia exhibited heightened vulnerability. Conidia in both control and native Chit46-treated groups displayed normal germination characteristics, including typical germ tube elongation (Fig. 3D). In contrast, conidia exposed to CBM-fused chitinases demonstrated abnormal germination patterns, characterized by swelling, distortion, and ultimately, cell lysis. This lysis effectively prevented their development into functional mycelia, highlighting the susceptibility of germinating conidia to CBM-fused chitinases. The rapid lysis of germinating conidia by CBM-fused variants, despite the resistance of mature conidia, may be attributed to the hydrolysis of nascent chitin being synthesized during germ tube formation and the transition to hyphal growth. Collectively, these observations provide a potential mechanistic explanation for why native Chit46 supports the growth and development of *T. harzianum*, whereas CBM-fused chitinases, with their enhanced ability to lyse self-structures, inhibit these processes.

Chitinases play crucial, multifaceted roles in fungal biology, including cell wall recycling, remodeling during growth, and the degradation of host cell walls during mycoparasitism. Maintaining a delicate balance is essential to ensure that the fungus’s own cell wall is protected from its secreted chitinases while effectively targeting host fungi. The mechanisms by which mycoparasitic fungi distinguish between self- and non-self-cell walls remain largely unexplored. Native Chit46, a CBM-free chitinase, is a pivotal invasive enzyme secreted by *Trichoderma* during mycoparasitism. In contrast, CBMs have been shown to be critical for the antifungal activity of other chitinases, such as chitinase C from *Streptomyces griseus* against *Trichoderma reesei*, where CBM deletion resulted in a 90% reduction in activity (45). In this study, exogenous native Chit46 did not harm *T. harzianum* but instead supported its growth. This suggests that native Chit46 is highly adapted to the physiological needs of *Trichoderma*, effectively enhancing its fitness within the constraints of self-tolerance while maintaining the ability to target host chitin. Conversely, exogenous CBM-fused chitinases caused significant damage to *T. harzianum* hyphae and germinating conidia, indicating that the presence of a CBM can lead to excessive or misdirected chitinase activity, resulting in self-inflicted cell wall damage. This potential for self-damage may represent an evolutionary constraint, offering a compelling rationale for why certain fungal chitinases, particularly those involved in mycoparasitism like Chit46, have not evolved or have lost CBMs. This effect is further underscored by prior findings that a chitinase-binding domain fusion to Chit42 elevated its activity against *T. harzianum* cell walls eightfold compared to the native enzyme (46). The absence of a CBM appears to be a strategic adaptation, allowing fungi to avoid self-harm while maintaining effective host degradation.

This contrasts with organisms lacking chitin in their own cell walls, such as bacteria and plants, whose chitinases typically contain one or more CBMs (47, 48). The intricate interplay between chitinases and fungal cell walls is thus illuminated, emphasizing the critical role of the presence or absence of CBMs in modulating chitinase activity and specificity. Interestingly, many lysozymes—glycosidases that target bacterial cell walls—also lack CBMs. This parallel observation suggests a broader biological principle where enhanced activity provided by substrate-binding modules may not always confer an evolutionary advantage, as it can lead to unintended consequences such as self-damage in organisms possessing similar cell wall polymers.

### Computational prediction of QID74–chitinase interactions suggests a protective mechanism

To elucidate the mechanisms by which *Trichoderma* protects its cell wall from endogenous chitinolytic attack, we investigated QID74, a conserved cell wall protein essential for resisting lytic enzymes (22). Although previous studies hypothesized that QID74 provides protection by binding to either cell wall chitin or chitinases directly, experimental evidence has been lacking. Given the technical challenges of modeling polysaccharide–protein interactions, we first explored the potential for QID74 to bind directly to the major chitinase, Chit46, using molecular docking simulations. Docking analyses consistently predicted a high-affinity interaction between QID74 and the substrate-binding pocket of Chit46 across all modeled conformations, with an average binding energy of −9.56 kcal/mol. This strong predicted interaction suggests that QID74 forms a stable complex with Chit46, potentially competitively inhibiting its hydrolytic activity against cell wall chitin. We further investigated how the fusion of CBMs to Chit46 might influence this interaction. Interestingly, docking simulations with CBM-fused Chit46 variants resulted in increased (less favorable) binding energies with QID74 (ranging from −2.60 to −7.30 kcal/mol) compared to native Chit46. This suggests that the presence of a CBM may sterically hinder or otherwise reduce the affinity of Chit46 for QID74, potentially compromising the protective function of QID74.

**Fig 4:**
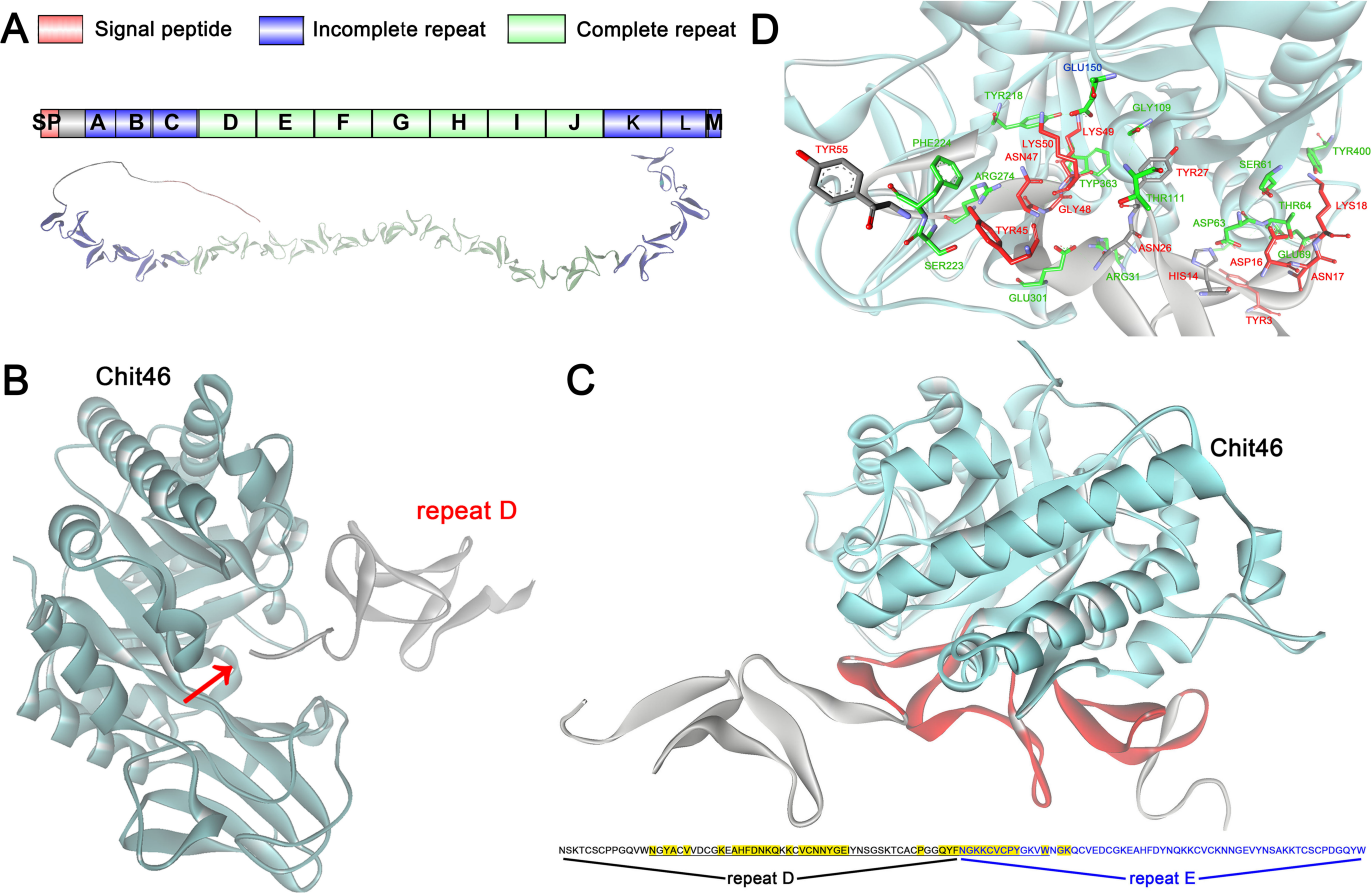
Simulation docking of repeating unit in QID74 with chitinase Chit46. (**A**) The amino acid sequence structure of QID74 and its predicted 3D structure from AlphaFoldDB (O74567). (**B**) Simulation docking of repeat D with Chit46. (**C**) Molecular docking between repeat DE and Chit46. Blue structure represents Chit46; red structure represents the interacting part of repeat DE; yellow highlighted sequence represents interacting residues. (**D**) Interfacing residues between repeat DE and Chit46. Red amino acid indicates the residue from QID74; green amino acid indicates the residue from Chit46.

To pinpoint the specific binding interface, we focused on the repetitive structure of QID74. Previous studies identified tandem 59-residue repeats (Fig. 4A) hypothesized to arise from shorter 13-residue motifs (21). Initial docking simulations using a single 59-residue repeat (unit D) showed its C-terminus inserting into the (α/β)_8_ TIM-barrel catalytic core of Chit46 (Fig. 4B), with a calculated binding energy of −6.22 kcal/mol. This interaction mode suggested that a single repeat might be insufficient for optimal binding. Indeed, docking a construct composed of two tandem repeats (DE) resulted in significantly enhanced affinity for the Chit46 substrate-binding pocket (Fig. 4C), with a more favorable binding energy of −7.82 kcal/mol. Detailed interface analysis of the Chit46-repeat DE complex revealed an extensive interaction network stabilized by 22 predicted hydrogen bonds and four salt bridges. Notably, 37 residues within the repeat DE construct directly contacted Chit46 (Fig. 4D). Crucially, the 35 residues specifically interacting with the substrate-binding pocket spanned a sequence length of exactly 59 amino acids, aligning perfectly with the length of a complete repeat unit in QID74. This striking alignment prompted a re-evaluation of the fundamental repeating unit in QID74.

Each of the newly defined complete repeats contains eight conserved cysteine residues and encompasses all residues predicted to interact with the Chit46 substrate-binding pocket. Furthermore, we determined that these new 59-residue units (herein designated repeats a–m) are assembled from smaller, conserved tridecapeptide motifs (consensus sequence NGKQCVCPKGQVW) (Fig. S3). Each 59-residue repeat contains two to four variations of this tridecapeptide motif, occasionally separated by one to two amino acids and linked by conserved DY or DS dipeptides. Phylogenetic analysis confirmed that full-length QID74, its novel 59-residue repeat units, and the underlying tridecapeptide motifs form distinct clades, underscoring their unique conservation within the genus *Trichoderma* (Fig. S4 to S6). The architecture of these cysteine-rich tandem repeats bears a structural resemblance to fungal LysM effectors, which bind chitin to evade plant chitinases during host colonization (49). By analogy, our findings suggest that QID74 employs a parallel strategy of direct protein–protein interaction, binding to endogenous chitinases like Chit46 to inhibit their activity and protect the *Trichoderma* cell wall from self-hydrolysis.

### Truncated QID74 inhibits chitinase activity via direct protein–protein interaction

**Fig 5:**
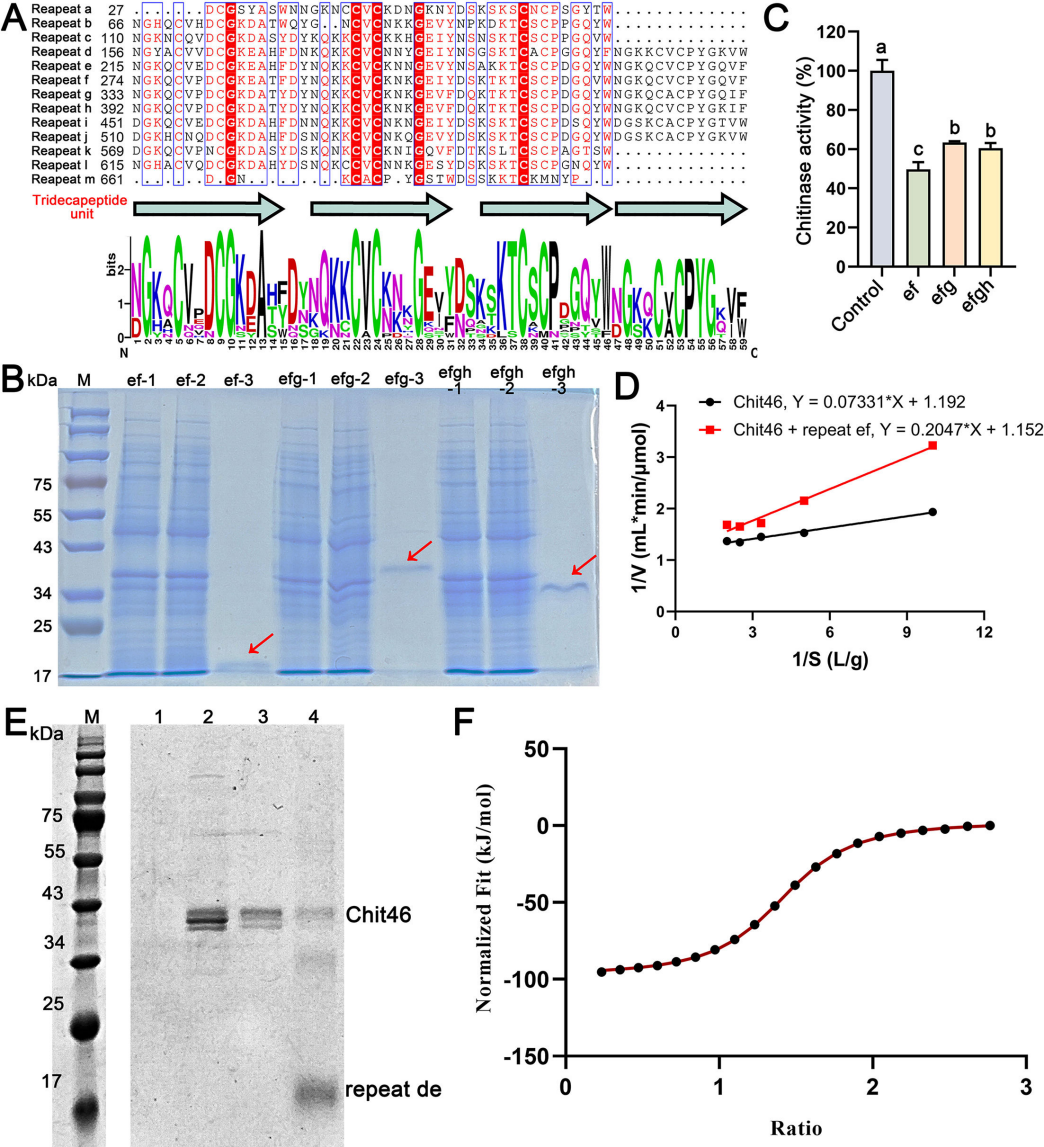
Molecular interaction between QID74 and chitinase Chit46. (**A**) Schematic representation of new modular architecture of QID74, highlighting the 59-residue repetitive units (arrows indicate conserved tridecapeptide motifs). (**B**) Heterologous expression and purification of QID74 repetitive domains (ef, efg, and efgh) in *Escherichia coli*. Lanes: 1, uninduced control; 2, induced cell lysate; 3, purified recombinant proteins. (**C**) Effects of QID74 repetitive domains on Chit46 enzymatic activity using colloidal chitin as substrate. Data represent mean ± SD (*n* = 3). (**D**) Lineweaver–Burk plot of Chit46 kinetics in the presence of the QID74-ef domain, indicating competitive inhibition. (**E**) His pull-down assay validating direct binding between Chit46 and repeat ef. Lanes: 1, wash fraction of the bait protein (repeat ef); 2, flow-through fraction of the prey protein (Chit46); 3, wash fraction of the prey protein; 4, eluted complex; (**F**) Isothermal titration calorimetry profiling of Chit46-repeat ef interaction showing fitted binding isotherm.

To elucidate the biochemical mechanism of QID74-mediated protection, we sought to characterize its interaction with the primary chitinase, Chit46. Despite successful expression of QID74 in *Saccharomyces cerevisiae*, this protein was located in yeast cell wall with low expression level, and only faint bands were detectable via Western blot (22), rendering it challenging for biochemical analysis. Therefore, the heterologous expression of QID74 in *E. coli* and *Pichia pastoris* was investigated. Despite numerous efforts, the production of recombinant QID74 was unsuccessful with natural or optimized codons. This recalcitrance likely stems from the protein’s intrinsically disordered regions and its highly repetitive, long amorphous structure. Given these setbacks, we turned to express truncated versions of QID74, composed of varying numbers of 59-residue repeats. Only the proteins containing two to four units (ef, efg, and efgh) were effectively expressed (Fig. 5B). Upon induction, engineered *E. coli* produced substantial amounts of proteins. Subsequent purification yielded homogeneous protein, which was clearly discernible as a single band on SDS-PAGE. The apparent molecular weights of purified proteins were larger than the theoretical values for unclear reasons, except for the repeat ef proteins, whose molecular weights were consistent with expectations (~20 kDa).

Biochemical assays using chitin powder as a substrate revealed that all truncated variants significantly inhibited Chit46 activity, with the ef variant exhibiting the highest inhibition rate at 52% (Fig. 5C). To define the nature of this inhibition, kinetic analysis was performed. Double-reciprocal (Lineweaver–Burk) plots showed lines intersecting at the *y*-axis, demonstrating that truncated QID74 acts as a competitive inhibitor. Specifically, the addition of the inhibitor increased the *K_m_* from 0.062 to 0.178 g/L, while the *V*_max_ remained largely unaffected (0.84–0.87 μmol/min/L). This competitive mode of action indicates that QID74 directly targets the substrate-binding pocket of Chit46. Notably, the inhibitory effect on CBM-fused chitinases was markedly reduced (below 25%, Fig. S7), supporting our previous docking predictions that CBM fusion hinders QID74 binding. Furthermore, truncated QID74 variants showed no measurable adsorption to chitin powder or colloidal chitin. This critical finding demonstrates that QID74 does not safeguard the cell wall by masking the chitin substrate, but rather by acting as a “shield” protein that directly binds and neutralizes self-chitinases.

The physical interaction between truncated QID74 and Chit46 was confirmed via a His pull-down assay and ITC. In the pull-down assay, the His-tagged “repeat de” served as the bait. The absence of Chit46 in the final wash of the resin-only control confirmed that the target protein does not non-specifically adhere to the cobalt resin (Fig. S8). Conversely, the presence of distinct bands for both Chit46 and repeat de in the experimental eluate indicates a stable protein–protein complex (Fig. 5E). Quantitative binding affinity was determined through ITC (Fig. 5F), which yielded a dissociation constant *K*_*D*_ of 5.39 μM. This value characterizes the interaction as one of moderate strength, providing sufficient affinity to inhibit chitinolysis while potentially allowing for a dynamic association during cell wall remodeling. The thermodynamic profile revealed a stoichiometry (*N*) of 1.36, indicating a likely 1:1 binding ratio with some variability due to the repetitive nature of the motifs. The large negative enthalpy (Δ*H* = −95.34 kJ/mol) and negative entropy change (Δ*S* = −219.07 J/mol·K) suggest that the interaction is primarily driven by hydrogen bonding and van der Waals forces, consistent with the predicted formation of extensive hydrogen bond networks in our docking models. These results provide the first direct biochemical evidence that *Trichoderma* employs QID74 as a specialized proteinaceous inhibitor to prevent self-inflicted damage during its mycoparasitic offensive.

### Potential mechanism of *Trichoderma* for protecting cell walls from self-chitinases

The fungal response to various stressors is intricately linked to the modification of cell wall components, where carbohydrate-binding proteins serve a crucial role. Recent studies have highlighted how LysM effectors contribute to the protective barrier of fungal cell walls by mitigating degradation from exogenous plant chitinases (50). Various chitin-binding proteins identified within the fungal cell wall provide a protective cushion against plant cell wall-degrading enzymes, interactions that are vital for fungal survival under enzymatic stress (49, 51, 52).

Fungal cell wall protective proteins, such as QID74, were previously hypothesized to function similarly to carbohydrate-binding proteins by either influencing the induction of cell wall-degrading enzymes or directly masking chitin from degradation (18). Rather than binding to chitin, QID74 appears to interact directly with chitinases, likely due to hydrophobic properties that mimic the enzyme’s natural substrate. Critically, the natural absence of a CBM in Chit46 emerges as a key adaptive feature that enhances its regulatory dependency on QID74. While a CBM typically confers high-affinity binding to insoluble chitin, its absence in Chit46 likely reduces the enzyme’s intrinsic avidity for the cell wall, rendering it more susceptible to modulation by soluble regulators like QID74. This structural characteristic may be integral to the fungus’s ability to finely control chitinase activity at the cell wall, preventing uncontrolled self-degradation. This revelation suggests that QID74 is primarily involved in modulating chitinase activity rather than forming a passive physical barrier like LysM.

QID74 is a member of the SCREPs, some of which protect fungal cell walls from hydrolysis by plant chitinases (53). Recent research has emphasized plant SCREPs, such as OsRMC, which inhibit fungal enzymes from *Magnaporthe oryzae* to enhance rice defense strategies (54). *Trichoderma* species possess a high number of small SCREPs in their genomes (55), while *in silico* predictions revealed a high content of small SCREPs, which, along with saccharide-degrading enzymes and proteins with unknown function, comprise over 60% of the secretomes (56). Structural cell surface proteins with this characteristic have in common their ability to form extracellular protein–protein complexes, some of which maintain a cell barrier (57).

The response of *Trichoderma* to chitin involves a complex interplay of regulatory mechanisms and enzymatic activities. When glucose is replaced with chitin as the sole carbon source—a condition that mimics mycoparasitic behavior—both *ech42* and *qid74* are significantly upregulated (15, 22). Previous investigations into the *qid74* promoter have uncovered a MYC2-like regulatory region situated near the start codon. This sequence, along with other MYC elements, is characteristic of genes associated with mycoparasitism, particularly those linked to chitin degradation, such as *chit42* and *papA* (58). The presence of these regulatory elements indicates a functional role for QID74 in chitin degradation processes, suggesting that it plays a significant role in the coordinated response of *Trichoderma* to hosts or their cell wall components. This response is multifaceted, involving the synthesis of various hydrolytic enzymes and QID74. This dual strategy may serve to mitigate the risk of cell wall degradation that could arise from the excessive activity of hydrolytic enzymes. While the absence of QID74 increases sensitivity to exogenous lytic enzymes but not its own (22), *Trichoderma* likely possesses additional self-protective mechanisms. For instance, hydrophobins—which are also SCREPs with a conserved eight-cysteine pattern—may contribute to protective functions (20). Given that hydrophobins are produced by all filamentous fungi, the protective mechanism of QID74 may exhibit species specificity to avoid protecting host fungi during an attack. Sequence alignment supports this, showing QID74 is highly conserved within *Trichoderma* species but shares less than 50% homology with non-*Trichoderma* species.

**Fig 6:**
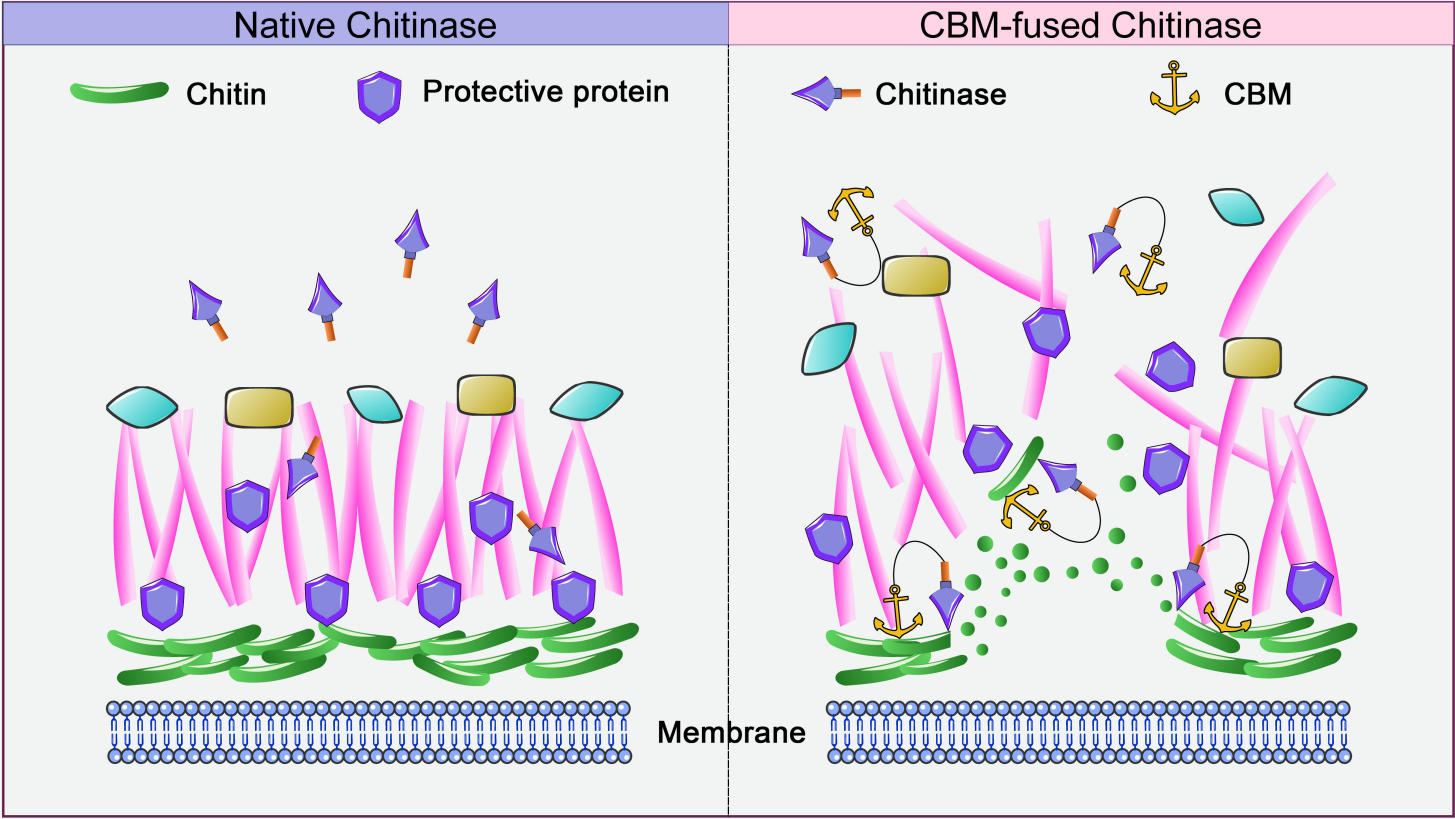
The possible arrow-shield model in *Trichoderma harzianum* of how carbohydrate-binding modules (CBMs) enhance the attack ability of chitinase on the self-fungal cell wall. (Left) Normally, the protective proteins QID74 reversibly bind the chitinase in the self-fungal cell wall, making chitin inaccessible for CBM-lacking group A chitinases; (right) CBM-fused chitinases have a stronger substrate-binding affinity for chitin, reduce the effect of protective proteins, and cause fungal cell walls to be destroyed.

In this “arrow-and-shield” model, QID74 acts as a shield for cell wall chitin by binding to the “arrows” (Chit46) to inhibit enzymatic activity (Fig. 6). CBM fusion significantly enhances the substrate affinity of Chit46 (25), disrupting this regulation by allowing the enzyme to bypass QID74 and penetrate the cell wall. This provides strong reverse evidence for the adaptive advantage of the natural CBM-deficient state in maintaining cellular integrity.

Insights into the structural characteristics and functional processes of fungal chitinases and their interaction with cell wall proteins at various developmental stages enable a deeper understanding of their roles in *Trichoderma* biology. Besides, a molecular evolutionary analysis of chitinases suggests that group A chitinases are likely more involved in growth and development rather than direct mycoparasitism (59). Conversely, group B chitinases show a strong association with mycoparasitic and entomopathogenic functions, while group C chitinases appear to be linked with host range expansion among certain plant-pathogenic fungi in the Sordariomycetes. However, recent findings indicate a potential overlap, as group C chitinases may not be distinctly resolved from group A (60). Thus, the biological functions of *Trichoderma* chitinases are intricately complex and extend beyond mere mycoparasitism, highlighting the necessity for further research to elucidate the multifaceted roles these enzymes play within the fungal kingdom. In summary, the co-evolution of a CBM-deficient chitinase (Chit46) and its regulatory SCREP (QID74) may represent a sophisticated adaptive strategy in *Trichoderma*. This system allows the fungus to precisely control the activity of a key hydrolytic enzyme deployed during mycoparasitism, minimizing the risk of self-harm while maintaining offensive capability. This fine-tuned regulation likely contributes significantly to the ecological fitness and success of *Trichoderma as* a mycoparasite.

### Conclusion

This study elucidates the essential role of the cell wall-associated protein QID74 in safeguarding *T. harzianum* from endogenous enzymatic degradation by chitinase Chit46. The natural absence of CBMs in group A chitinases is a critical evolutionary adaptation. The incorporation of CBMs disrupts the established arrow-and-shield balance, allowing the enzymes to bypass QID74-mediated protection and hydrolyze the fungal self-cell wall. This nuanced regulation of protein–protein interactions between QID74 and CBM-free chitinases appears fundamental to self-recognition and cellular integrity in *Trichoderma*. Furthermore, the potent cell wall-destructive capabilities of engineered CBM-fused Chit46 variants offer a promising biotechnological strategy for the control of recalcitrant plant pathogenic fungi. Finally, the characterization of QID74 as a protective shield suggests that similar SCREPs in other organisms may serve as viable candidates for enhancing host resistance against chitinases.

## SUPPLEMENTAL MATERIAL

Supplemental material mbio.00018-26-s0001.docx Fig. S1-S8; Table S1.

ASM does not own the copyrights to Supplemental Material that may be linked to, or accessed through, an article. The authors have granted ASM a non-exclusive, world-wide license to publish the Supplemental Material files. Please contact the corresponding author directly for reuse.
