# Supplementary material for: Protein QID74 protects the cell wall of Trichoderma from degradation caused by its own chitinase, which lacks a carbohydrate-binding module
Source: mBio. 2026 Apr 20;17(5):e00018-26. doi: 10.1128/mbio.00018-26 (PMC13170347; doi:10.1128/mbio.00018-26)
Supplement: Supplemental material — Fig. S1-S8; Table S1. [file mbio.00018-26-s0001.docx]

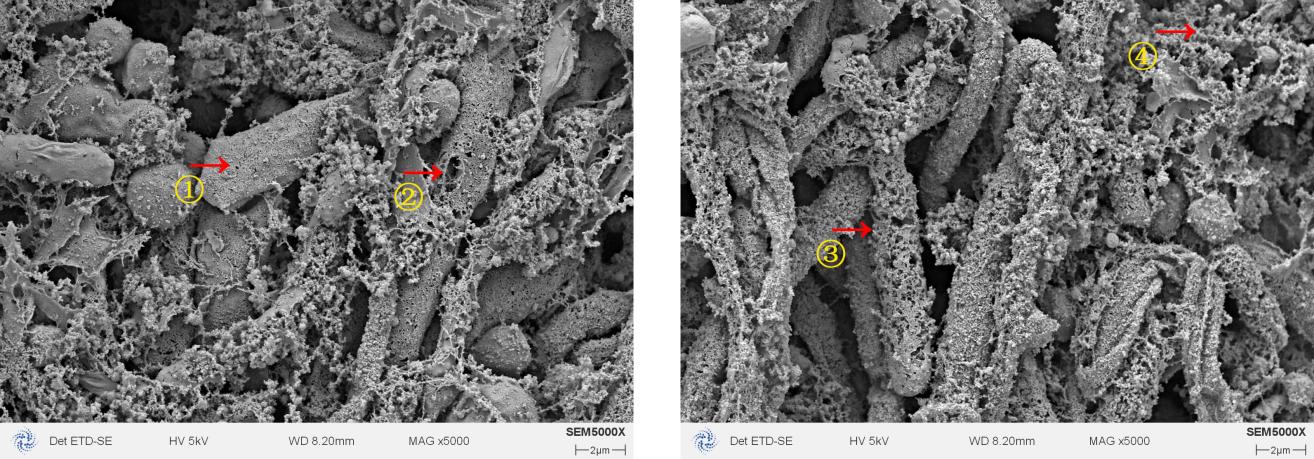


**FIG S1.** The potential process by which Chit46-CBM3 disrupts the cell wall of *Trichoderma*.


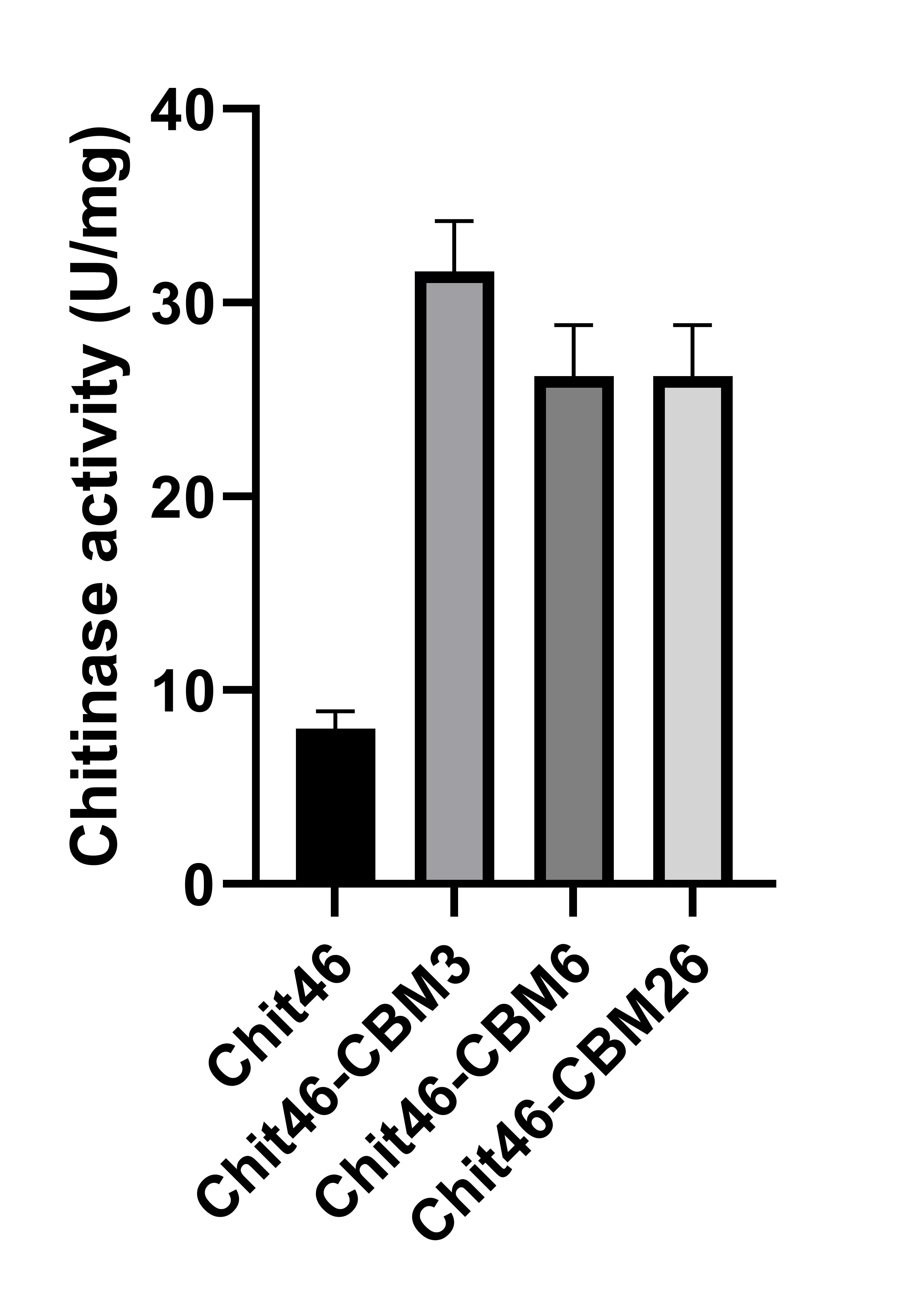


**FIG S2.** Substrate specificities of native and modified Chit46 for *T. harzianum* cell walls.


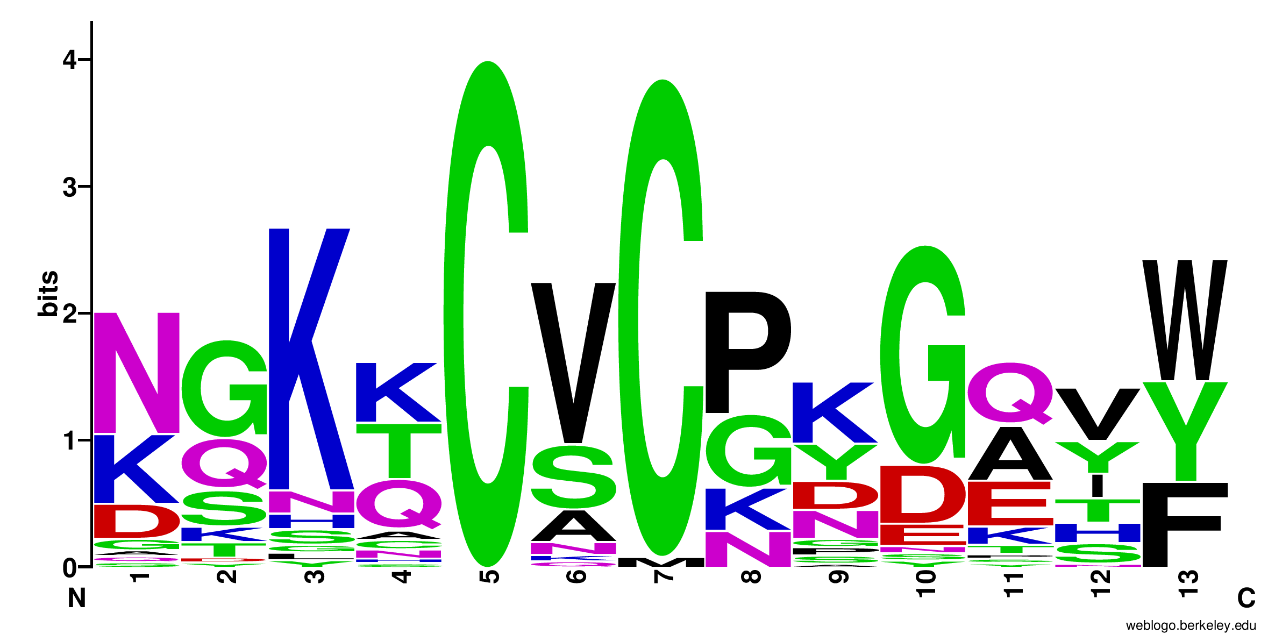


**FIG S3.** Sequence logos of tridecapeptide units in QID74.


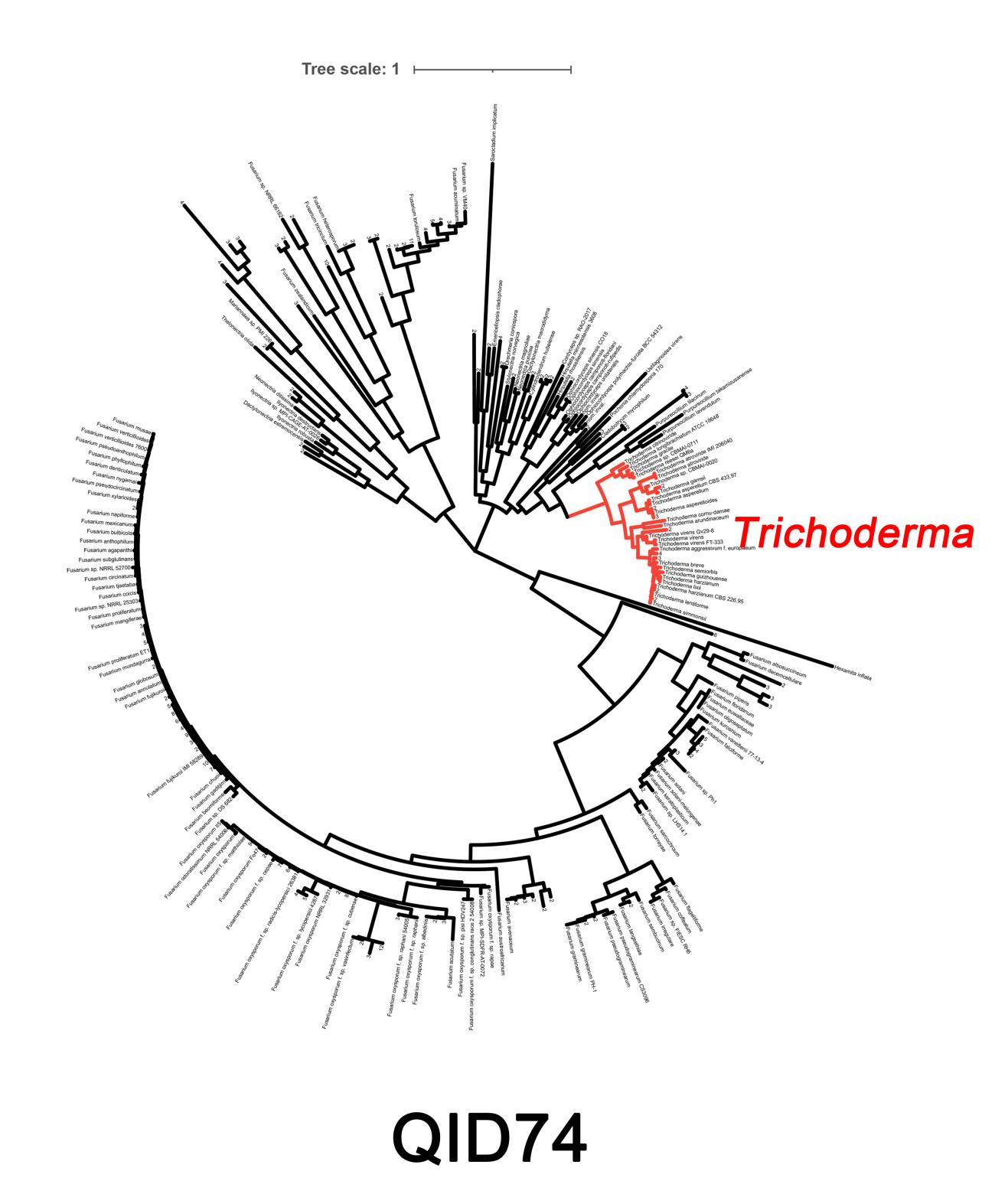


**FIG S4.** Phylogenetic analysis of the QID74 protein. Proteins from *Trichoderma* were marked in red.


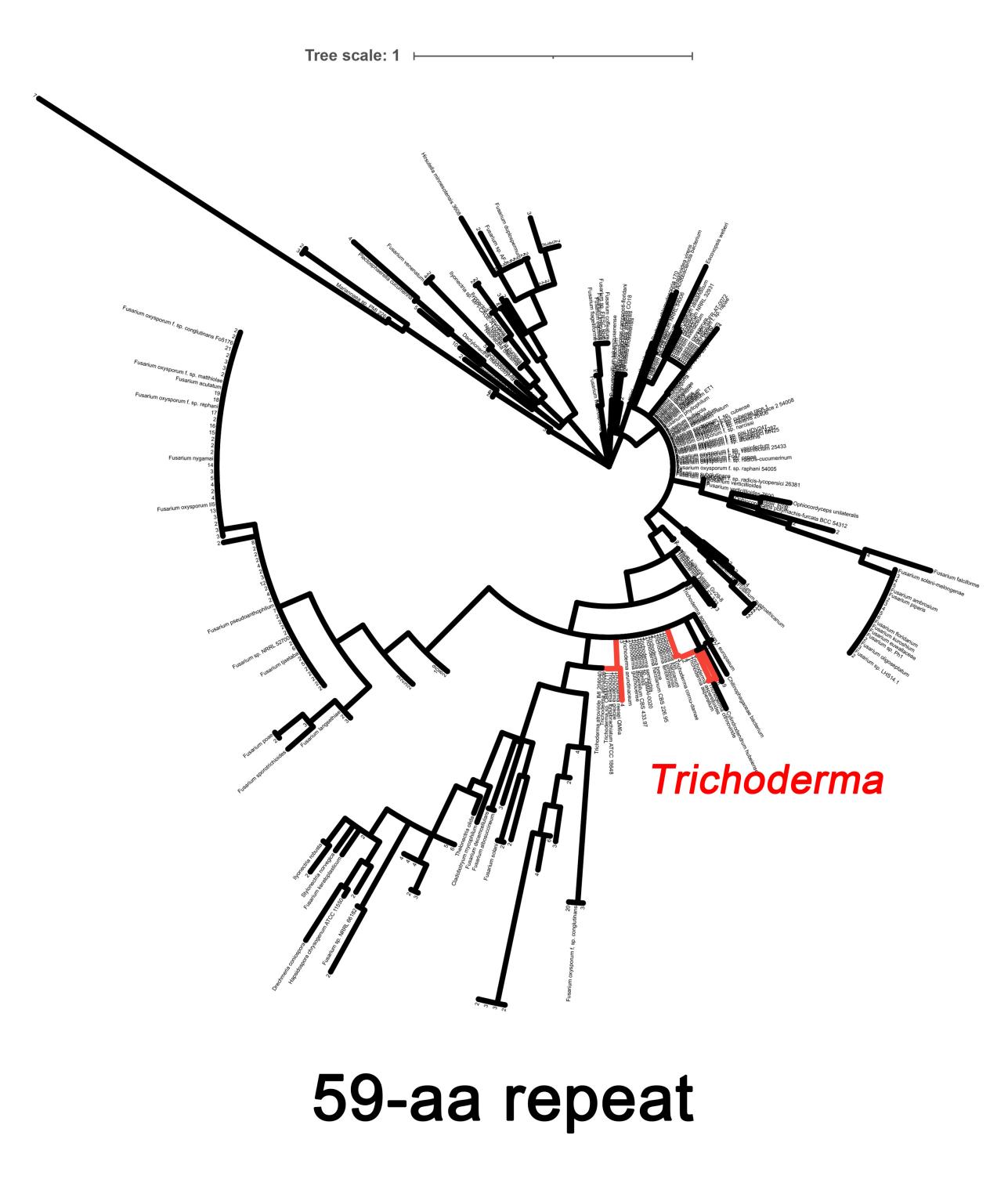


**FIG S5.** Phylogenetic analysis of the 59-aa repeat protein. Proteins from *Trichoderma* were marked in red.


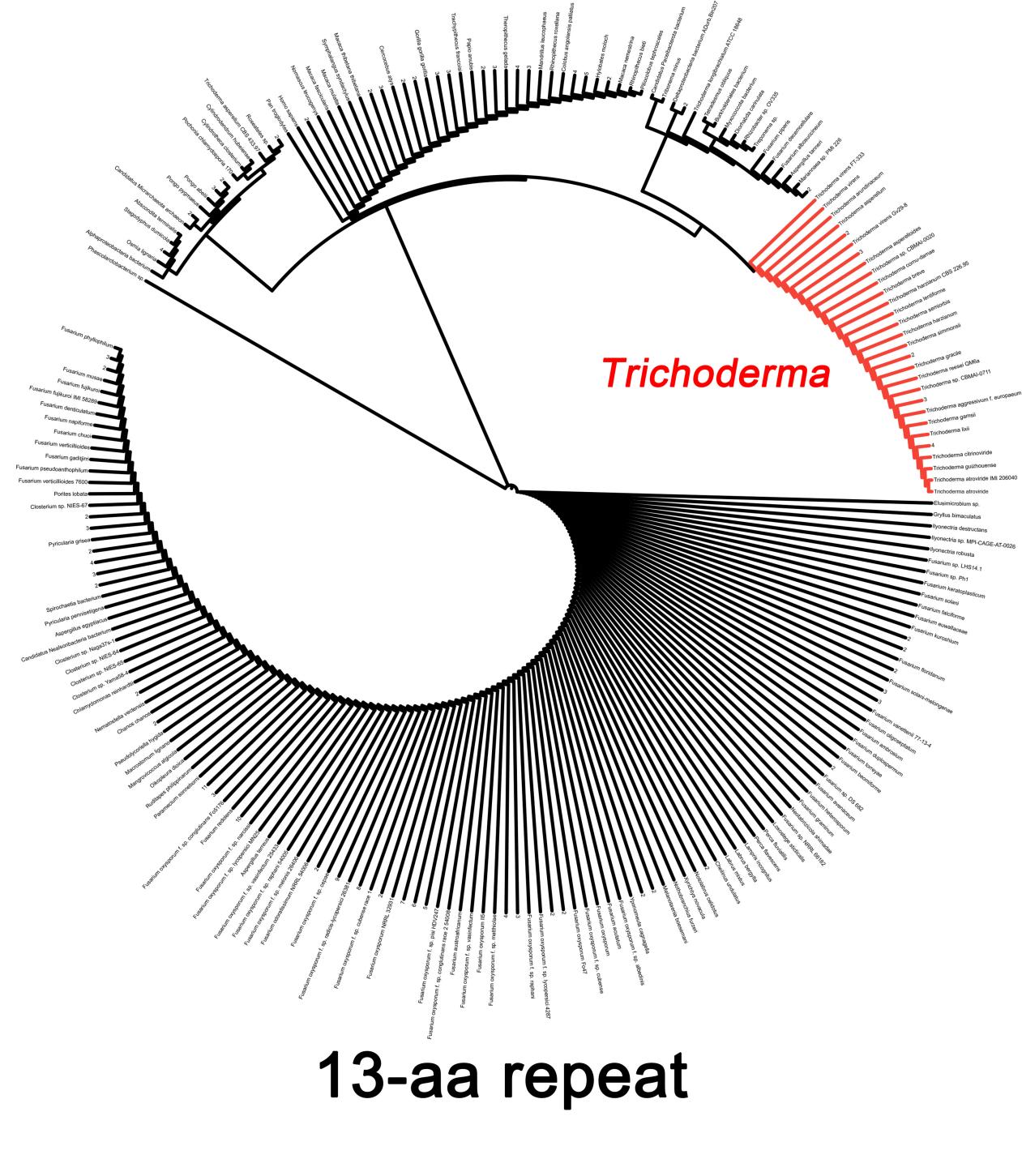


**FIG S6.** Phylogenetic analysis of the 13-aa repeat protein. Proteins from *Trichoderma* were marked in red.


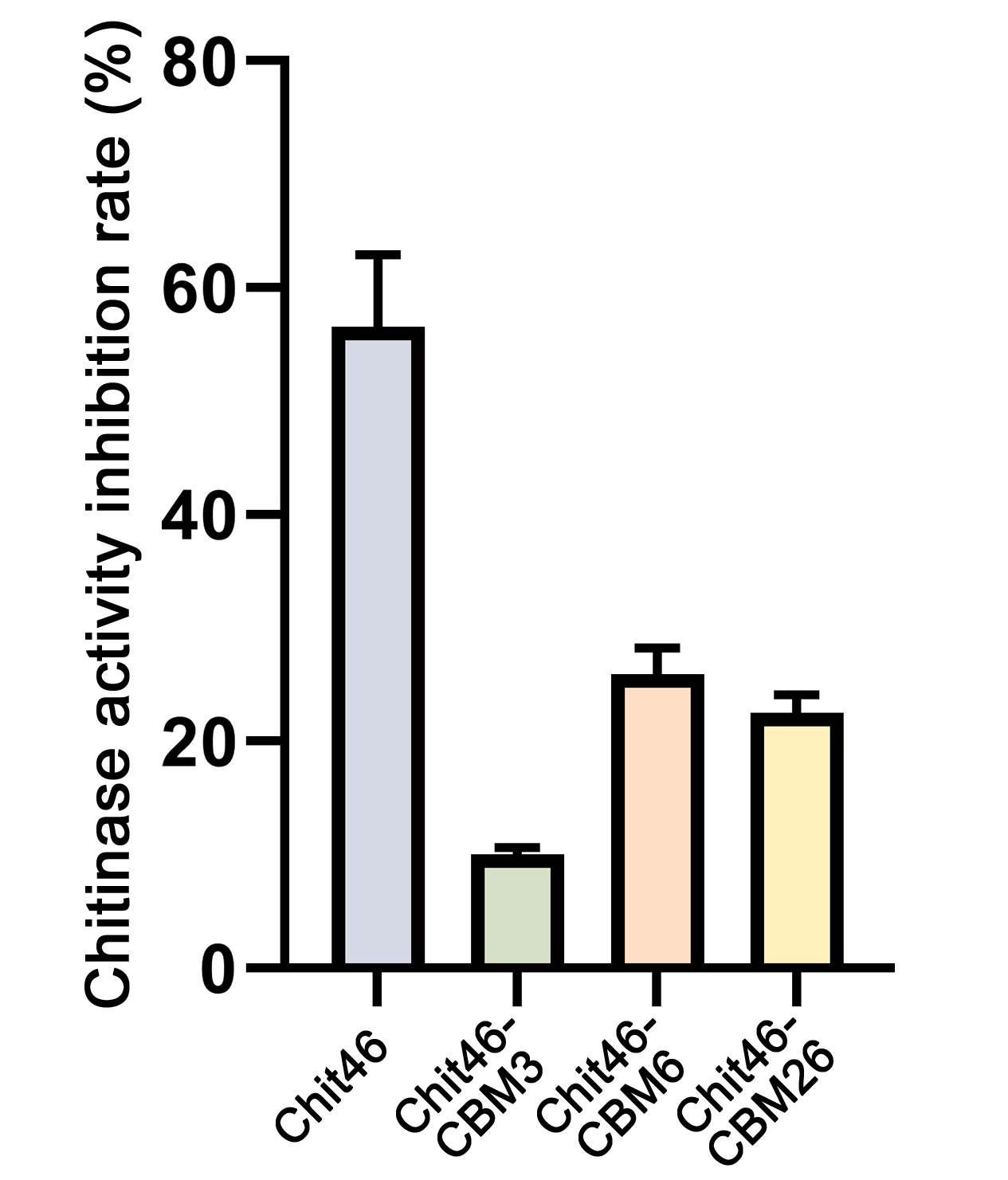


**FIG S7.** The effects of repeat ef from QID74 on chitinases activity of Chit46 and CBM-fused chitinase using chitin powder as the substrate.


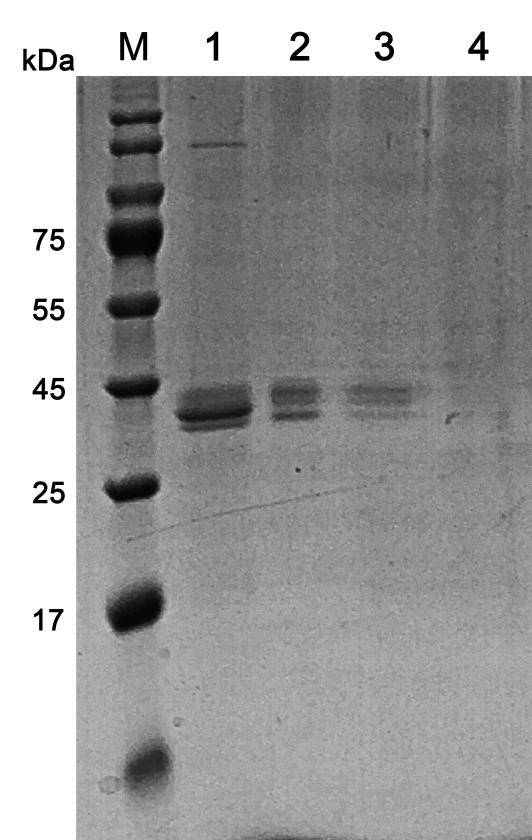


**FIG S8.** His pull-down assay validating non-specific binding of Chit46. Lanes: 1, prey protein (Chit46); 2, Chit46 input; 3, Chit46 flow-through; 4, Chit46 elution.

**Table S1. Primers used in this study.**

| Primer | Sequence (5’-3’) |
| --- | --- |
| Repeat-f2 | TGGGTCGCGGATCCGAATTCAACGGTAAACAGTGTGTAGAAGACT |
| Repeat-r1 | caagcttgtcgacggagctcGAAGACTTGGCCGTACGG |
| Repeat-r2 | CAAGCTTGTCGACGGAGCTCGAAGACCTGGCCGTACGG |
| Repeat-r3 | caagcttgtcgacggagctcGAAGATCTGACCGTATGGACAGG |
